# Differential Regulation of Bone Marrow-Derived Endothelial Progenitor Cells and Endothelial Outgrowth Cells by the Notch Signaling Pathway

**DOI:** 10.1371/journal.pone.0043643

**Published:** 2012-10-31

**Authors:** Jing-Yuan Chen, Lei Feng, Hai-Long Zhang, Jun-Chang Li, Xin-Wei Yang, Xiu-Li Cao, Li Liu, Hong-Yan Qin, Ying-Min Liang, Hua Han

**Affiliations:** 1 Department of Hematology, Tangdu Hospital, Fourth Military Medical University, Xi'an, People's Republic of China; 2 State Key Laboratory of Cancer Biology, Department of Medical Genetics and Developmental Biology, Fourth Military Medical University, Xi'an, People's Republic of China; Leiden University Medical Center, The Netherlands

## Abstract

Endothelial progenitor cells (EPCs) are heterogeneous populations of cells that participate in vasculogenesis and promote tissue regeneration. However the different roles of EPC populations in vasculogenesis and tissue regeneration, as well as their regulation and mechanisms remain elusive. In the present study, we cultured bone marrow (BM)-derived early EPCs (EEPCs) and endothelial outgrowth cells (EOCs), and investigated their roles in liver regeneration and their regulation by the Notch signaling pathway. We found that Notch signaling exhibited different effects on the proliferation and migration of EEPCs and EOCs. Our results also showed that while EEPCs failed to form vessel-like structures in a three dimensional sprouting model in vitro, EOCs could sprout and form endothelial cords, and this was regulated by the Notch signaling. We further showed that, by using a conditional knockout model of RBP-J (the critical transcription factor mediating Notch signaling), Notch signaling differentially regulates EEPCs and EOCs. In a partial hepatectomy (PHx) model, EEPCs Notch-dependently benefitted liver regeneration with respect to liver function and hepatocyte proliferation and apoptosis. In contrast, EOCs appeared not directly involved in the recovery of liver function and the increase of hepatocytes. These data suggested that the RBP-J-mediated Notch signaling differentially regulated the two types of EPCs, which showed different roles in liver regeneration.

## Introduction

Endothelial progenitor cells (EPCs) are progenitor cells derived from mesodermal progenitor cells in early embryogenesis, and are responsible for initial vascularization in both embryo body and extra-embryonic tissues through a process defined as vasculogenesis [1], [2]. In the past decade it has been recognized that EPCs also exist in adult tissues, mostly in bone marrow (BM), and take part in neovascularization at the sites of ischemia in disease models. EPCs can be mobilized from BM and can home to wounded tissues [3], [4], where they can differentiate into endothelial cells (EC) to directly participate in vasculogenesis, and/or to produce angiogenic factors to contribute to vascular remodeling. Moreover, a large body of evidence has suggested that EPCs have therapeutic benefits in the treatment of ischemic diseases [5]. For example, several groups have shown the roles of EPC in liver regeneration and in the therapy of liver cirrhosis [6], [7].

However, the effects of EPCs on the repair of tissue damages appear varied as reported by researchers in different sets of preclinical and clinical studies [8]. This inconsistency is at least partially attributable to the heterogeneous nature of EPCs [9]. EPCs in BM or just entering the peripheral blood express stem cell markers such as CD34 and CD133, together with VEGFR2 (KDR). Along with in vitro culturing and maturation, the cells gradually lost stem cell markers, and begin to express EC-specific antigens such as platelet endothelial cell adhesion molecule 1 (PECAM-1 or CD31) and VE-cadherin, among others [10]. Other researchers have suggested that EPCs is composed of endothelial lineage cells at different differentiation stages [11]. Two types of EPCs have been identified from in vitro cultured EPCs, which are supposed to have different cellular origins [12], [13]. Early EPCs (EEPCs) are spindle-like in shape, and have limited proliferative potential and can be cultivated no more than 4 weeks in vitro. Endothelial outgrowth cells (EOCs) or late EPCs, in contrast, have a cobblestone-like appearance and maintain a high proliferative potential. EEPCs are myeloid endothelial progenitor cells, originating from CD14^+^ monocytic cells, while OECs are derived from CD14^−^ cells. But further defining different subpopulations of EPCs and understanding their roles and mechanisms in vascularization is still required.

EOCs and EEPCs can be involved in the formation of new blood vessels through different mechanisms such as differentiating into ECs or producing angiogenic cytokines [14]–[17]. Signals regulating their mobilization and functions have been elusive. Among the molecules identified so far, such as angiogenic factors [18], integrins [19] and adhesion molecules [20], the stroma-derived factor (SDF)-1α-CXCR4-mediated signaling plays an important role in the trafficking and the homing of EPCs [21]–[25]. SDF-1α induced by hypoxia inducible factor (Hif)-1α enhances the adhesion, migration, and homing of circulating CXCR4-positive EPCs to ischemic tissues [22], [26]. Another important signaling pathway in EPCs is the Notch receptor-mediated signaling. The Notch pathway is highly conserved in evolution, and plays an essential role in cell fate determination in multiple lineages of stem and progenitor cells [27]. There are five Notch ligands (Jagged1, 2, and Delta-like [Dll]1, 3, 4) and four Notch receptors (Notch1–4) in mammals. Ligand binding triggers proteolytic cleavages of Notch receptors, releasing the Notch intracellular domain (NICD) to translocate into the nucleus, where NICD associates with the transcription factor RBP-J and recruits other co-activators to activate target gene expression [28]. Kwon et al [29] have shown that the Jagged1-mediated Notch signaling promote adult neovascularization by regulating the function of EPCs. We have also found that Notch-RBP-J signaling regulates the mobilization, migration and function of EPCs through the expression of CXCR4 [30]. However, the roles of the Notch signaling pathway in different subpopulations of EPCs, namely EEPCs and EOCs, have not yet been revealed. In this study, we accessed this question by using in vitro cultured EPCs and RBP-J knockout mice. Our data have suggested that the Notch-RBP-J signaling regulates the functions of EEPCs and EOCs in different ways.

## Results

### Characterization of in vitro cultured EEPCs and EOCs

**Figure 1:**
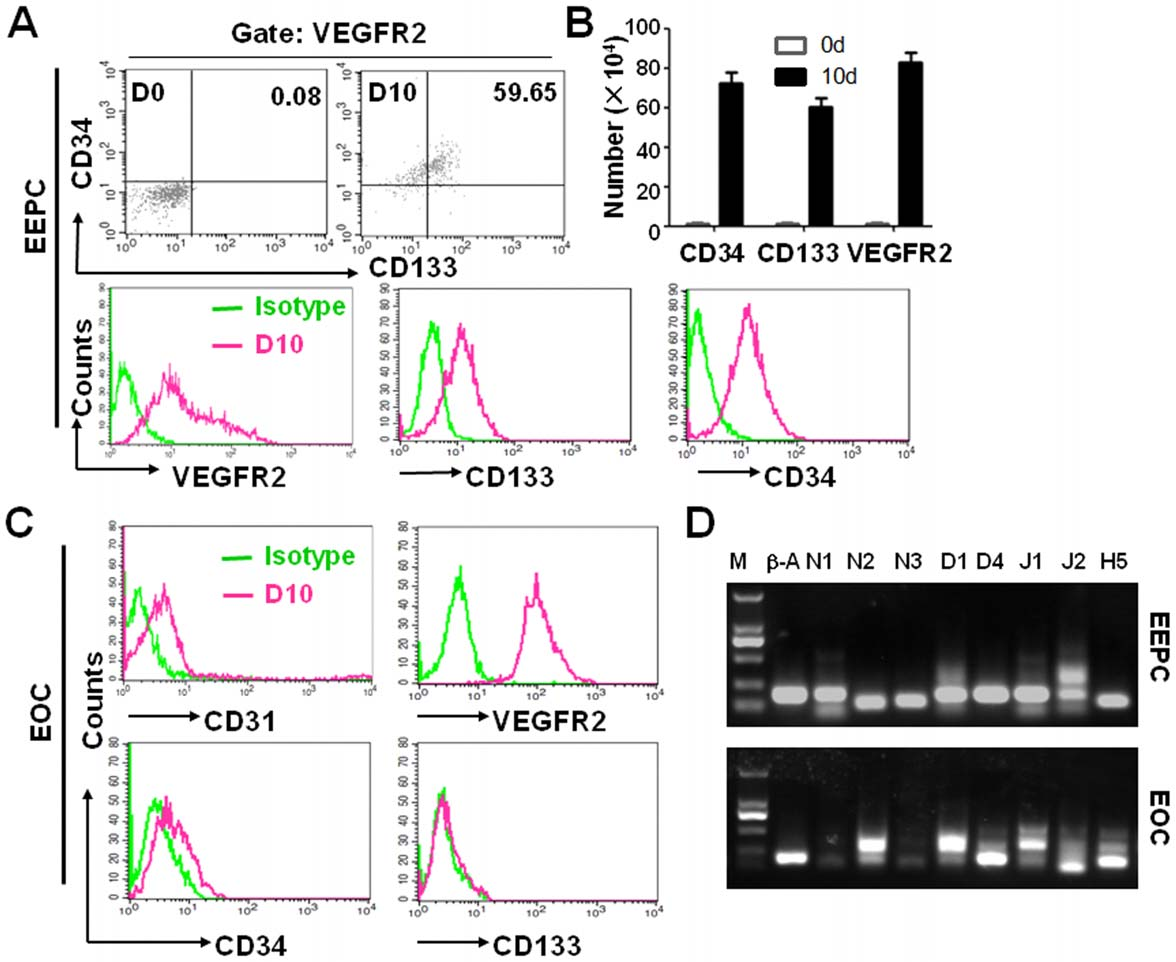
Differential expression of Notch-related molecules in BM-derived EEPCs and EOCs. (**A**) BM mononuclear cells were cultured under conditions to generate EEPCs. Cells that were freshly isolated (D0) or cultured for 10 days (D10) were labeled with fluorescent antibodies to CD133, CD34, and VEGFR2, and were analyzed by FACS. (**B**) The numbers of cell in (**A**) were calculated and shown. (**C**) The EEPC culture in (**A**) was continued for 8 more weeks to generate EOCs. Cells were stained with fluorescent antibodies against CD133, CD34, VEGFR2 and CD31, and were analyzed by FACS. (**D**) At the end of the cultures in (**A**) and (**C**), cells were collected, and the expressions of Notch (N) 1, 2, 3, Dll (D) 1, 4, Jagged (J) 1, 2 and Hes (H) 5 were examined by using RT-PCR, with β-actin as a reference control.

In freshly isolated BM mononuclear cells, cells with the EEPC phenotypes (CD34^+^/CD133^+^/VEGFR2^+^) accounted for only 0.08% of total population of cells. After being cultured for 6 days in the EPC medium, this percentage reached 8.95%, and kept increasing up to 50.59% on day 10 of the culture (Figure 1A, upper panels). The absolute number of cells increased in a similar way (Figure 1B). The increase of cell percentage was accompanied by remarkable up-regulations of VEGFR2, CD133 and CD34 (Figure 1A, lower panels). Under microscope, these cells had a spindle-like shape, consistent with the phenotypes of EEPCs [31].

In contrast to the spindle-like EEPCs, EOC culture generated cells with a cobblestone appearance after being cultured for 6–8 weeks (Figure S1). These cells expressed mature EC markers CD31/VEGFR2 but did not express the progenitor cell markers CD133 and CD34 (Figure 1C). These phenotypes were consistent with EOCs [32].

We preliminarily examined the expression of the Notch signal-related genes in EEPCs and EOCs by using reverse transcription-polymerase chain reaction (RT-PCR). The results showed that most of the genes we tested were expressed in both EEPCs and EOCs, but the two types of EPCs had different expression patterns in the Notch signal-related molecules (Figure 1D).

### Blocking Notch signaling showed different effects on the proliferation and migration of EEPCs and EOCs

**Figure 2:**
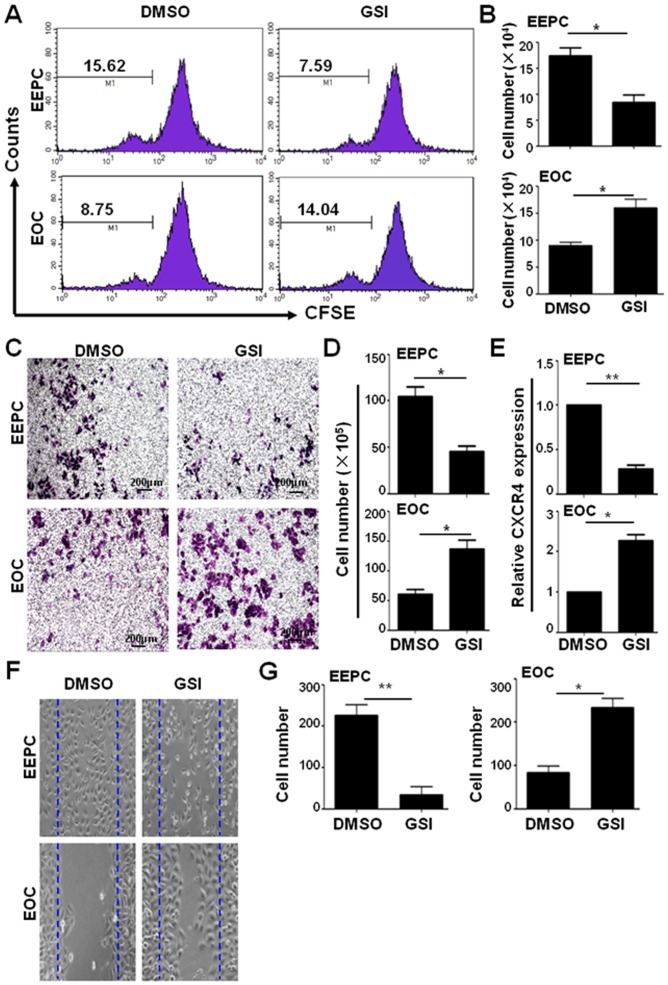
Blockade of Notch signaling showed different effects on the proliferation and migration of EEPCs and EOCs. (**A**) EEPCs and EOCs were cultured as above, labeled with CFSE and were cultured further in the presence of DMSO or GSI for 48 h. Cell proliferation was analyzed by FACS. (**B**) The absolute number of proliferating cells were calculated and compared. (**C**) Migration assay. Fourteen hours post migration, cells attached to lower membrane were fixed (4% paraformaldehyde) and stained with 0.1% crystal violet (Magnifications, ×10). (**D**) Cells in (**C**) were counted and compared statistically. (**E**) The expressions of CXCR4 in EEPCs and EOCs were analyzed by real time RT-PCR. (**F,G**) Cell scratch assay. EEPCs and EOCs were seeded and cultured to confluence. Scratches were made in each culture, and the edges of the scratches were marked by lines. Cells were further cultured in the presence of DMSO or GSI for 2 more days for the observation of cell migration. The numbers of cells migrating into the areas between the lines were counted and compared statistically (**G**). Bars = mean ± SD, n = 3, *P<0.05, **P<0.01.

To evaluate the role of the Notch signaling pathway in EEPCs and EOCs, we treated these cells with a γ-secretase inhibitor (GSI) to block Notch signaling. EEPCs and EOCs were pre-labeled with carboxyfluorescein diacetate succinimidyl ester (CFSE), and cell proliferation was examined by fluorescence-activated cell sorter (FACS) on the fifth day (EEPCs) or second day (EOCs), due to the different proliferating rate between the two sub-populations, after the addition of GSI into the culture. As shown in Figure 2A, blocking the Notch signaling pathway in EEPCs resulted in significantly decreased cell proliferation. However, in contrast, blocking Notch signaling increased the proliferation of EOCs (Figure 2A, lower). Direct cell counting revealed the same results: Notch blockade led to decreased number of EEPCs but increased number of EOCs after culture (Figure 2B).

We then assessed the effect of Notch blockade on the migration of EEPCs and EOCs by using a transwell assay. EEPCs and EOCs were cultured in the presence of GSI or DMSO for 72 h. And cells were seeded at a density of 1.5×10^5^ per well in the upper compartment and were cultured at 37°C for 14 h. Cells in the lower membrane were counted. The results showed that blocking of Notch signaling by GSI led to decreased migration of EEPCs in response to SDF-1α, whereas the same treatment resulted in increased migration of EOCs in response to SDF-1α (Figure 2C and 2D). Previous data including ours have shown that Notch signaling regulated EPC mobilization most likely through dynamic modulation of CXCR4 expression [30]. We therefore examined the expression of CXCR4 in EEPCs and EOCs in the presence of GSI. The results showed that the expression of CXCR4 in EEPCs was reduced in the presence of GSI. But in contrast, the expression of CXCR4 mRNA in EOCs was up-regulated upon blocking Notch signaling pathway by GSI (Figure 2E). We also assessed the effect of Notch blockade on the migration of EEPCs and EOCs by using the cell scratch assay. EEPCs and EOCs were cultured to confluence and a scratch was made in each culture. Cells were cultured further in the presence of GSI, and cells migrating into the scratched areas were counted. The results showed that blocking of Notch signaling by GSI led to decreased migration of EEPCs (226±15.1 in control vs. 33.3±11 in GSI-treated) (*P*<0.01), whereas the same treatment resulted in increased migration of EOCs (83.3±8.8 in control vs. 233.3±12 in GSI-treated) (*P*<0.05) (Fig. 2F and 2G). These results indicated that Notch signaling played opposite roles in the proliferation and migration of EEPCs and EOCs.

### Notch signal blockade led to increased sprouting and endothelial sprout extension by EOCs

**Figure 3:**
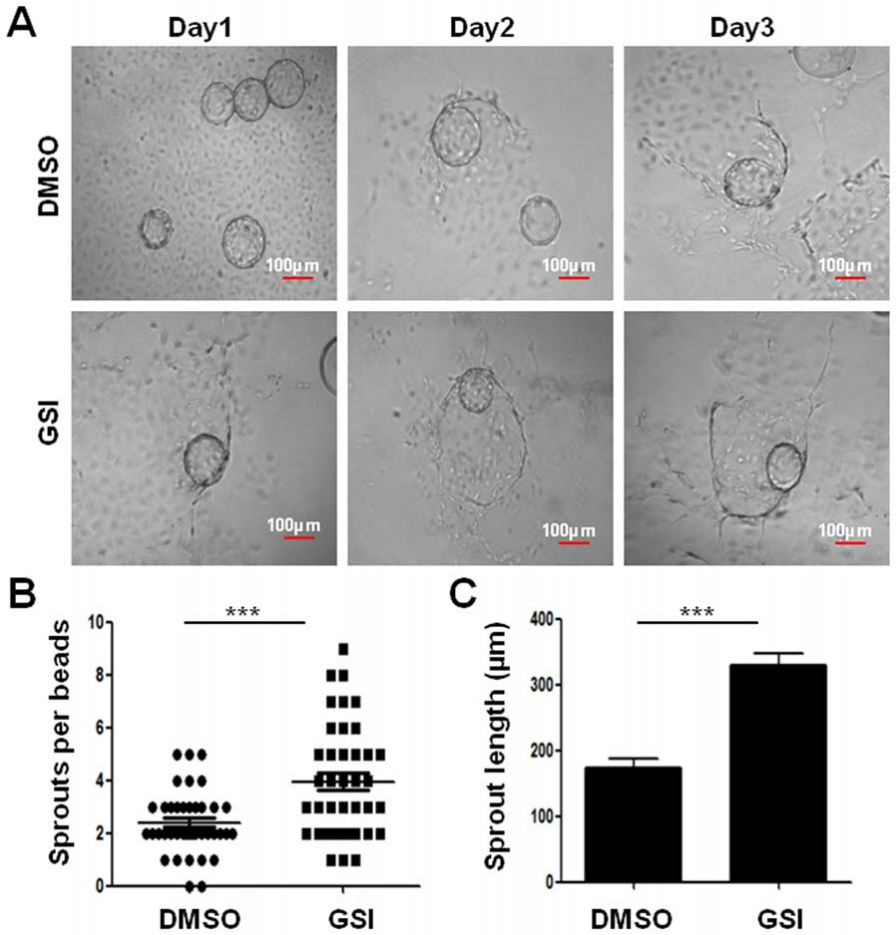
Blockade of Notch signaling increased the sprouting and the endothelial cord extension by EOCs. (**A**) Cytodex 3 microcarrier beads were coated with EOCs and were incubated in fibrinogen clots in the presence of DMSO or GSI. Images of the beads were captured by an inverted microscope on different days of culture. (**B**) Comparison of the number of sprouts per beads between the two groups. (**C**) The length of endothelial sprouts extending from each bead was measured and compared between groups. Totally 77 beads from each group were analyzed. Bars = mean ± SD, ***P<0.001.

We next evaluated the ability to form vessels by EEPCs and EOCs by using a three dimensional in vitro sprouting model, in which cells were attached to Cytodex 3 microcarrier beads and were permitted to sprout in fibrinogen gels [33]. EEPCs failed to sprout (data not shown). When EOCs were cultured in the system, sprouting started on around day 2, and cord-like sprouts grew out with the culture being proceeded (Figure 3A; Figure S2). In the presence of GSI, the number of the sprouts and the length of the sprouts were significantly increased as compared with the control (Figure 3A–3C). This result suggested that blocking the Notch signaling pathway could promote the ability of EOCs to participate in vessel formation, likely through increased sprouting and endothelial sprout extension.

### RBP-J deficient EEPCs and EOCs displayed different tendency of homing into liver during liver regeneration

**Figure 4:**
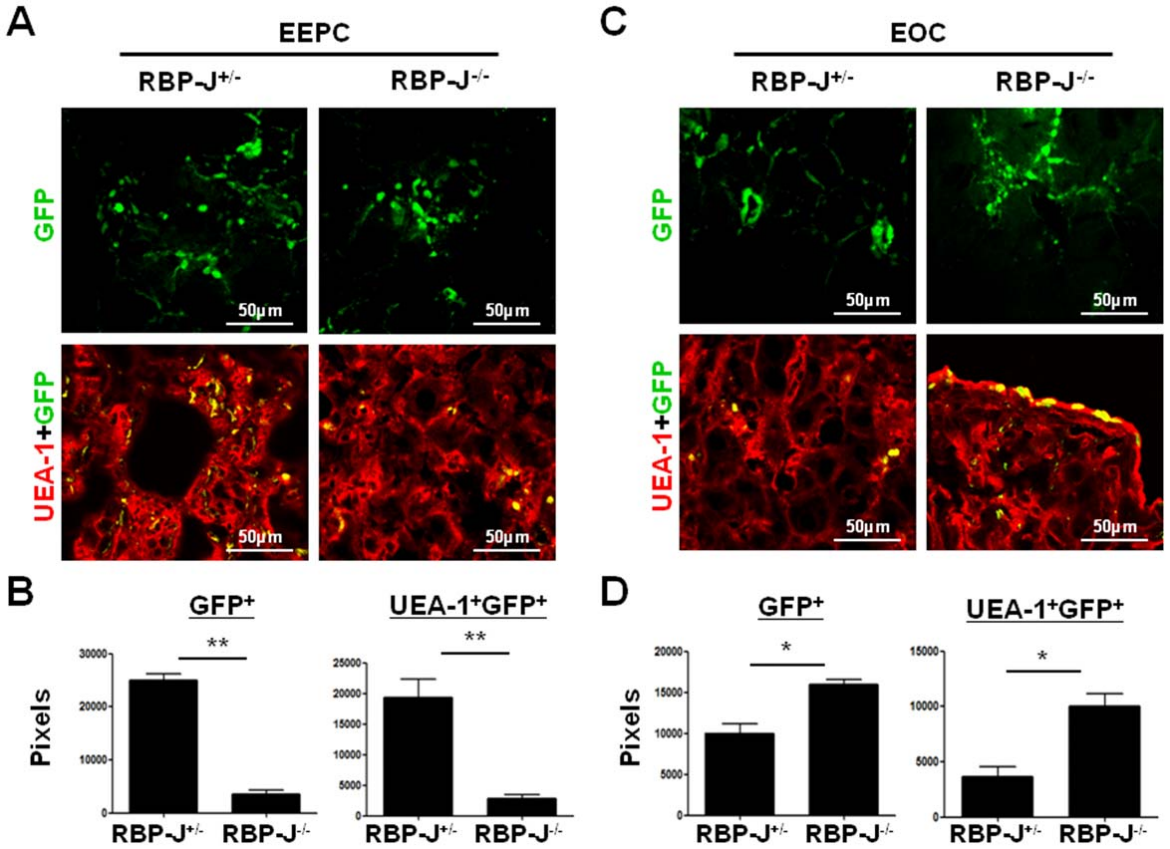
RBP-J deficient EEPCs and EOCs display different ability to home into liver during Phx-induced liver regeneration. Normal mice were subjected to PHx. On the day of the operation, mice were transfused through the tail veins with EEPCs (**A**, **B**) or EOCs (**C**, **D**) derived from GFP^+^RBP-J^−/−^ or GFP^+^RBP-J^+/−^ mice. Five days after the transplantation, the livers of the recipient mice were sectioned and stained, and were examined under a fluorescence microscope for GFP^+^ cells and UEA-1^+^GFP^+^ cells (**A**, **C**). GFP^+^ cells and UEA-1^+^GFP^+^ cells were quantitatively represented by corresponding pixels (**B**, **D**). Bars = mean ± SD, n = 4, *P<0.05, **P<0.01.

EPCs could migrate to injured tissues and participate in tissue repair and regeneration through various mechanisms [3]. We have shown that EPCs participate in partial hepatectomy (PHx)-induced liver regeneration, and this role is regulated by Notch signaling [30]. Next we tried to clarify the role of Notch signaling in EEPCs and EOCs during liver regeneration induced by PHx. To achieve this, we employed the RBP-J conditional knockout mouse crossed with the Mx-Cre transgenic mouse, in which injection of poly(I)-poly(C) could induce almost 100% of RBP-J deletion in BM [34]. GFP^+^ EEPCs and EOCs derived from the RBP-J deficient and the control mice were transfused into irradiated wild type mice on the day of PHx. Five more days later mice were perfused, and GFP^+^ cells homing into the liver were examined under a fluorescence microscope. As shown in Figure 4A and 4B, EEPCs from RBP-J deficient mice appeared to home into the regenerating liver less efficiently compared with cell from the control mice. In contrast, the number of EOCs homing into the regenerating liver appeared increased upon RBP-J deletion (Figure 4C and 4D). These observations suggested that the disruption of RBP-J in EEPCs retarded their homing to liver whereas the same mutation promoted EOCs homing to the liver during liver regeneration.

### EEPCs but not EOCs promoted the regeneration of liver function and this was regulated by Notch signaling

**Figure 5:**
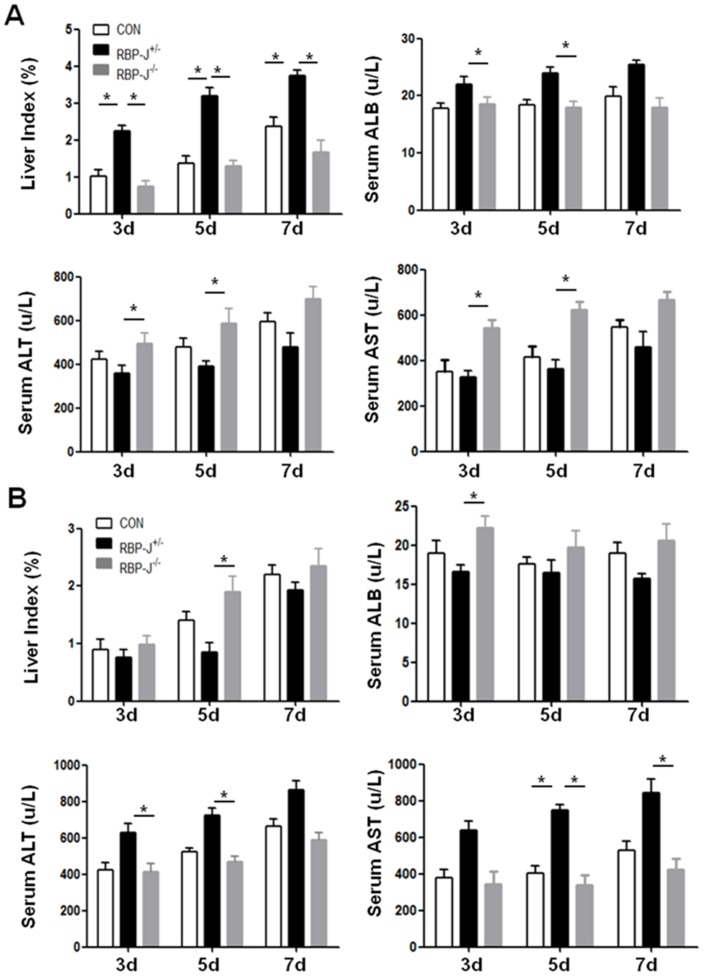
RBP-J deficient EEPCs and EOCs showed opposite effects on the liver regeneration after PHx. Normal mice were subjected to PHx. On the day of the operation, mice were transfused through the tail veins with EEPCs (**A**) or EOCs (**B**) derived from the RBP-J^−/−^ or the RBP-J^+/−^ mice. The control (CON) mice were transfused with PBS. Liver index, serum ALB, ALT and AST of the recipient mice were determined on day 3, 5 and 7 after the transfusion. Bars = mean ± SD; n = 4; *P<0.05.

In order to observe the effects of the transfused EEPCs and EOCs on the functional regeneration of liver after PHx, wild type irradiated mice were subjected to PHx. On the day of the operation, PBS, or EEPCs or EOCs derived from the RBP-J knockout or the control mice were transfused into the recipient mice. On day 3, 5 and 7, the recipient mice were tested for the serum alanine aminotransperase (ALT), aspartate aminotransferase (AST), and albumin (ALB), as well as liver index. The results showed that the transfusion of the control EEPCs significantly promoted the increase of liver index and serum ALB, but these effects were canceled by RBP-J deficiency in the transfused cells (Figure 5A). Transfusion of the RBP-J deficient EEPCs led to an increase in serum AST and ALT in the recipient mice (Figure 5A). In contrast to EEPCs, the transfusion of the control EOCs showed no beneficial or even some adverse effects on functional regeneration of livers after PHx (Figure 5B). Upon RBP-J deletion, the effects of EOCs in increasing serum ALS and AST were abrogated (Figure 5B). These observations suggested that while EEPCs but not EOCs showed beneficial effects on PHx-induced liver regeneration, the Notch-RBP-J signaling was essential for the liver regeneration-promoting function of EEPCs.

### EEPCs but not EOCs promoted the regeneration of hepatocytes, and was regulated by Notch-RBP-J signaling pathway

**Figure 6:**
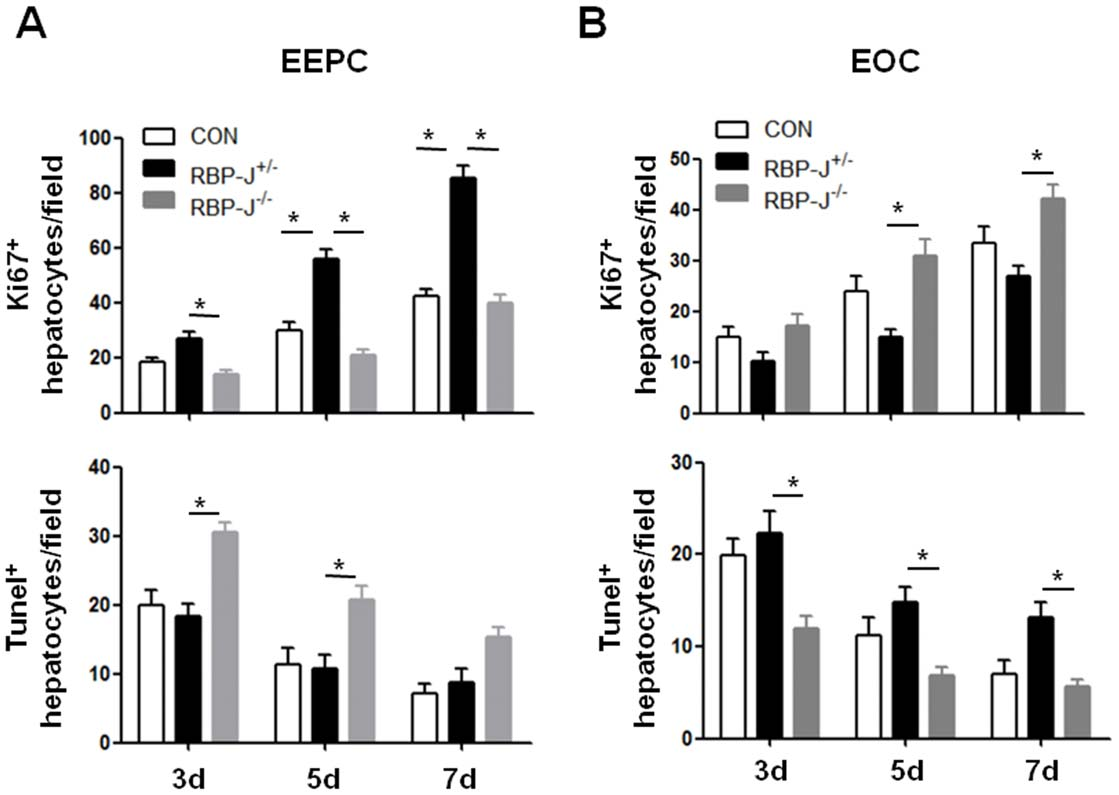
RBP-J deficient EEPCs and EOCs showed opposite effects on cell proliferation and apoptosis during liver regeneration after PHx. Mice were subjected to PHx and were transfused with EEPCs (**A**) or EOCs (**B**) derived from the RBP-J^−/−^ or the RBP-J^+/−^ mice as above. Cell proliferation and apoptosis in the livers of the recipient mice were determined on day 3, 5 and 7 after the transfusion by using anti-Ki67 staining and TUNEL (Figure S3, S4), respectively. Ki67^+^ round nuclei and TUNEL^+^ cells were counted under microscope, and were compared between groups. Bars = mean ± SD, n = 4, *P<0.05.

The functional regeneration of liver after PHx is dependent on the proliferation of hepatocytes. On day 3, 5 and 7 after transplantation of RBP-J^+/−^ EEPCs, the regenerating liver showed increased hepatocyte proliferation and decreased hepatocyte apoptosis. However, these effects were abrogated when the transfused EEPCs were RBP-J deficient (Figure 6A, Figure S3). In contrast to EEPCs, transplantation of EOCs from the control mice did not influence hepatocyte proliferation or their apoptosis, but RBP-J deficient EOCs showed a trend of increasing proliferation and decreasing apoptosis of hepatocytes (Figure 6B, Figure S4). These findings proposed that EEPCs and EOCs had different effects on PHx-induced liver regeneration, and that EEPCs benefited liver regeneration by promoting hepatocyte proliferation and reducing apoptosis, which could be regulated by the Notch-RBP-J signaling pathway.

## Discussion

EPCs are phenotypically heterogeneous cell populations with different origins. These cells express multiple surface molecules including CD14, CD45, CD31, CD105, CD146, VE-cadherin, and VEGFR2 [35], [36], some of which were shared by other types of cells such as monocytes and macrophages [37], [38]. CD133, CD34 and VEGFR2 as the classical markers of EPCs have been doubted recently [39]. Our study has shown that the CD34^+^CD133^+^VEGFR2^+^ population of adult BM-derived EPCs can be expanded in vitro and give rise to EOCs under the endothelial culture conditions, indicating that they represent a population of endothelial precursors. Moreover, EPCs also show functional heterogeneicity, such as their involvements in tissue repair and new vessel formation [40]. To clarify different roles of EEPCs and EOCs in vessel formation, we employed a three dimensional vessel sprouting model that could avoid some of the shortcomings of the Matrigel assay system [41]. Unlike the Matrigel assay, the three dimensional sprouting model provides the possibility to observe the initiation and extension of sprouts of ECs directly. We found that EOCs but not EEPCs could sprout and form endothelial cords as assayed in this system, in agreement with recent reports on these two subsets of EPCs. We further confirmed that blocking the Notch signaling pathway significantly increased sprouting by EOCs.

EEPCs and EOCs might play different roles in tissue repair and regeneration. Our transplantation experiments have shown that EEPCs can promote liver regeneration with respect to liver function and hepatocyte proliferation and apoptosis, although these cells appear incompetent in directly participating in vessel formation, at least in vitro. In contrast, EOCs could sprout and form vessel-like endothelial cords under appropriate conditions, but EOCs seem not be able to promote liver regeneration in our systems. Moreover, our results suggest that EEPCs and EOCs might take part in liver repair and regeneration through different mechanisms. EEPCs, which express high level of CXCR4, could be recruited to the site of tissue injury by the high level of SDF1α liberated by injured cells [24], [25], and participate in tissue repair and regeneration through paracrine factors [42]. EOCs, in contrast, expresses low level of CXCR4, are more destined to ECs and can participate in vessel formation likely through vasculogenesis (Figure S5). Blocking of Notch signaling differentially regulated CXCR4 expression in these two types of cells, likely resulting in their differential homing in the liver. Moreover, these cells might also be chemotracted to the injured tissues mainly by factors other than CXCR4, such as VEGF, which is highly induced by hypoxia through the Hif family transcription factors.

Our results showed that the RBP-J-mediated Notch signaling might be critical for the migration and function of both EEPCs and EOCs. Notch signaling pathway plays important roles in the colonization, self-renewal, migration and differentiation of EPCs [28]. Our recent study has shown that the Notch signaling pathway might regulate BM-derived EPCs and circulating EPCs differentially, and CXCR4 might play a critical role in these processes. The results reported here, by using in vitro cultured EEPCs and EOCs, are consistent with our previous data and have confirmed that Notch signaling plays differential roles in EEPCs and EOCs (Figure S5). EOCs represent more mature EPCs with respect to their lack of expression of the precursor cell surface antigens CD34 and CD133. The effect of Notch signaling on EOCs seems more similar to that on ECs, although EOCs can be distinguished from mature ECs by their appearance in in vitro culture and a much higher rate of proliferation [12], [43]. In addition to EPCs, Notch signaling also regulates the expression of CXCR4 in other cell types such as mature ECs [44] and dendritic cells [45]. However, the molecular mechanisms by which Notch signaling regulates CXCR4 have not been elucidated yet, leaving the differential regulation of CXCR4 expression in EEPCs and EOCs an open question.

## Materials and Methods

### Ethnic statements

The animal husbandry, experiments and welfare were conducted in accordance with the Detailed Rules for the Administration of Animal Experiments for Medical Research Purposes issued by the Ministry of Health of China, and were approved by the Animal Experiment Administration Committee of Fourth Military Medical University. Mice were raised in the specific pathogen free (SPF) conditions on the C57BL/6 background, and were manipulated with every specific care to reduce the suffering of the mice during the experiments.

### Mice

The RBP-J-floxed mice and the Mx-Cre transgenic mice were as described [34]. The RBP-J-floxed mice were crossed with the Mx-Cre mice to obtain heterozygous and homozygous mice bearing the Mx-Cre transgene (RBP^+/f^-MxCre and RBP^f/f^-MxCre, as the control and the RBP-J knockout mice, respectively), as genotyped by PCR [34]. The Cre-mediated deletion of RBP-J was induced by the intra-peritoneal injection of poly(I)-poly(C) (Sigma, St. Louis, MI) into 5-week-old mice with suitable genotypes for eight times as described [34]. PHx was performed as described previously [46].

### Culture of EEPCs and EOCs

BM mononuclear cells were obtained from 3-weeks-old male C57BL/6 mice. Total BM cells were suspended in HBSS (Invitrogen, Carlsbad, CA) and were overlaid onto Ficoll-Paque PLUS solution (Amersham Biosciences, Piscataway, NJ), and were centrifuged by using a swing-out rotor at 740 g for 30 min. Cells were collected from the interface, washed 3 times with the M199 medium (Invitrogen), and were resuspended in M199 supplemented with 20% fetal calf serum (FCS), 2 mM L-glutamine, 100 U/ml penicillin, 100 µg/ml streptomycin (Invitrogen), and 150 µg/ml endothelial cell growth supplement (ECGS, BD Biosciences, San Jose, CA), heparin (100 mg/ml), and 50 ng/ml human insulin-like growth factor-1 (IGF-1, Pepro Tech, Rocky Hill, NJ). The cells were seeded in 2% gelatin-coated 6-well plates at a density of 1×10^7^ cells/well, and were incubated at 37°C in 5% CO_2_-95% air in a humidified incubator. Non-adherent cells were removed 3 days later, and fresh medium was added. Cultures were maintained through day 10 and phenotype analysis of the cells was performed on days 0, 6 and 10.

For the culture of EOCs, mononuclear cells were resuspended in 12 mL M199 medium with the same supplements and were cultured under the conditions as above. Non-adherent cells were discarded 24 h later, and adherent cells were rinsed once with complete M199 medium, and fresh complete M199 medium was added to each well. First medium change was performed 3 days after the plating. Thereafter, medium was changed every 3 days until the first passage 4 weeks after the plating. Cells were cultured further until colonies of EOCs containing cobblestone-appearing cells appeared between 6 and 8 weeks of the culture.

### FACS analysis

Single cell suspensions were prepared from cultured or freshly isolated cells. Cells (3×10^5^) were stained with antibodies for 30 min on ice, and were analyzed by using a FACSCalibur™ (BD Immunocytometry Systems, San Jose, CA). Data were analyzed by using the CellQuest™ software. The antibodies and reagents used in FACS analyses included anti-mouse-CD133-FITC, anti-mouse-CD34-PE, biotinylated anti-mouse-Flk-1, streptavidin-APC and anti-CD31 (BD PharMingen, San Diego, CA). Dead cells were excluded by propidium iodide (PI) staining.

For cell proliferation assay, cells (1×10^6^ for EEPCs, 3×10^5^ for EOCs) were seeded in 6-well plates and were labeled with CFSE (Sigma) for 30 min. Cells were cultured for two more days, and cell proliferation was examined by FACS.

### Quantitative RT-PCR

Total RNA was extracted from cells using the TRIzol reagent (Invitrogen, Carlsbad, CA) according to the manufacturer's instructions. Complementary DNA was prepared by using a reverse transcription kit from TOYOBO (Osaka, Japan). Real-time PCR was performed by using a kit (SYBR Premix EX Taq, Takara) and the ABI PRISM 7300 real time PCR system, with β-actin as an internal control. Primers used in real time PCR were as follows: β-actin, CATCCGTAAAGACCTCTATGCCAAC and ATGGAGCCACCGATCCACA; CXCR4, GAAGTGGGGTCTGGAGACTAT and TTGCCGACTATGCCAGTCAAG.

### Migration assay

Chemotaxis experiments were performed in polycarbonate transwell inserts (8 µm pore, Corning Costar Corp.). SDF-1α (Peprotech) was added in the lower chamber at the concentration of 100 ng/ml. Cells were seeded at a density of 1.5×10^5^ per well in the upper compartment and were cultured at 37°C for 14 h. Non-migrating Cells were removed from the upper surface by gentle scrubbing. Migrating cells attached to the lower membrane stained with 0.1% crystal violet and were counted in five random fields.

### In vitro sprouting assay [32]

Cells were incubated with Cytodex 3 microcarrier beads (Sigma) at a ratio of 400 cells per bead in M199 medium containing 150 µg/ml ECGS at 37°C overnight. The cell-coated beads in PBS (0.5 ml) were adjusted with fibrinogen (Sigma) solution up to 2 mg/ml (200 beads/ml), and were added into one well of a 24-well plate containing 0.625 units thrombin (Sigma), followed by incubating for 5 min at room temperature and then at 37°C for 20 min to clot. The clots were equilibrated in M199 for 30 min at 37°C, and the medium was then replaced with fresh M199 medium. The cell-coated beads in clots were cultured for 5 days with medium change every other day. Images of the beads were captured by an inverted microscope, and the numbers of sprouts and the length of the endothelial sprouts were measured. Similar experiments were repeated in triplicates covering 400 photographs. In some cases GSI was added on the first day of the culture at the final concentration of 0.75 µM, with DMSO as a control.

### Immunofluorescence

Tissues embedded in OCT were sectioned at 10 µm thickness. For staining, sections were fixed with 4% paraformaldehyde and were stained with Rhodamine-UEA-l (Vector Laboratories, Burlingame, CA), or FITC-conjugated anti-Ki67 (Santa Cruz Biotechnology, Santa Cruz, CA). TUNEL was performed by using a kit (DeadEnd™ Fluorometric TUNEL System, Promega) according to the manufacturer's instructions. Images were taken under a fluorescence microscope (Olympus BX51, Japan) with a CCD camera, or a confocal microscope (FV1000, Olympus).

### BM transplantation

The femurs of mice were dissected and flushed with PBS. Total BM cells were treated with buffered 0.14 M NH_4_Cl for erythrolysis, and were resuspended at a density of 1×10^7^/ml. Wild type congenic mice as recipients were irradiated with 8 Gy of γ-ray. Cells (1×10^6^/ml) were transfused via tail vein. In some experiments, cells were collected from GFP transgenic mice, and were then transfused into the recipients. The mice were kept with water containing antibiotics (1.1 g/L of neomycin sulphate) until further analyses.

### Biochemistry

Serum ALT and AST were determined by using a Chemistry Analyzer (AU400, Olympus, Tokyo, Japan). Serum albumin was determined by using a kit (Roche, Basel, Swiss) with a Biochemical Analyzer (Roche).

### Statistics

The statistical analysis was performed with the SPSS 11.0 program. Results were expressed as the means ± SD. Comparison between groups was undertaken using the unpaired Student's t test. *P*<0.05 was considered statistically significant.

## Supporting Information

Figure S1 Culture of EEPCs and EOCs. EEPCs and EOCs were cultured as described in Materials and methods, and cells were photographed under a phase-contrast microscope. Magnifications, ×200.
(TIF)

Figure S2 Sprouting and tube formation of EOCs attached to Cytodex 3 microcarrier beads. For methods, see the Materials and methods section of the text. Beads are 70 to 150 µm in diameter.
(TIF)

Figure S3 RBP-J deficiency attenuated cell proliferation and increased apoptosis after the transfusion of EEPCs during liver regeneration after PHx. Mice were subjected to PHx and were transfused with EEPCs derived from the RBP-J^+/−^ or the RBP-J^−/−^ mice. Cell proliferation and apoptosis in the livers of the recipient mice was determined on day 3, 5 and 7 after the transfusion by using anti-Ki67 and TUNEL staining, respectively. Ki67^+^ round nuclei and TUNEL^+^ cells were counted under microscope. Comparison of the number of cells was shown in Figure 6A.
(TIF)

Figure S4 RBP-J deficiency attenuated apoptosis and increased cell proliferation after the transfusion of EOCs during liver regeneration after PHx. Mice were subjected to PHx and were transfused with EOCs derived from the RBP-J^+/−^ or the RBP-J^−/−^ mice. Cell proliferation and apoptosis in the livers of the recipient mice were determined on day 3, 5 and 7 after the transfusion by using anti-Ki67 and TUNEL staining, respectively. Ki67^+^ round nuclei and TUNEL^+^ cells were counted under microscope. Comparison of the number of TUNEL^+^ cells was shown in Figure 6B.
(TIF)

Figure S5 Differential regulations of EEPCs and EOCs by the Notch-CXCR4 signaling. Notch signaling increases homing of EEPCs in BM by the upregulation of CXCR4. In contrast, Notch signaling represses CXCR4 expression by EOCs, therefore reduces their homing to BM. EOCs can be recruited into injured tissues by other signals such as VEGF, and participate in vessel formation likely through vasculogenesis. Therapeutic transfusion of EEPCs can lead to recruitment of EEPCs into injured liver and participates in tissue repair and regeneration through paracrine effects.
(TIF)
