# Supplementary figures and images for: Differential Regulation of Bone Marrow-Derived Endothelial Progenitor Cells and Endothelial Outgrowth Cells by the Notch Signaling Pathway
Source: PLoS One. 2012 Oct 31;7(10):e43643. doi: 10.1371/journal.pone.0043643 (PMC3485270; doi:10.1371/journal.pone.0043643)

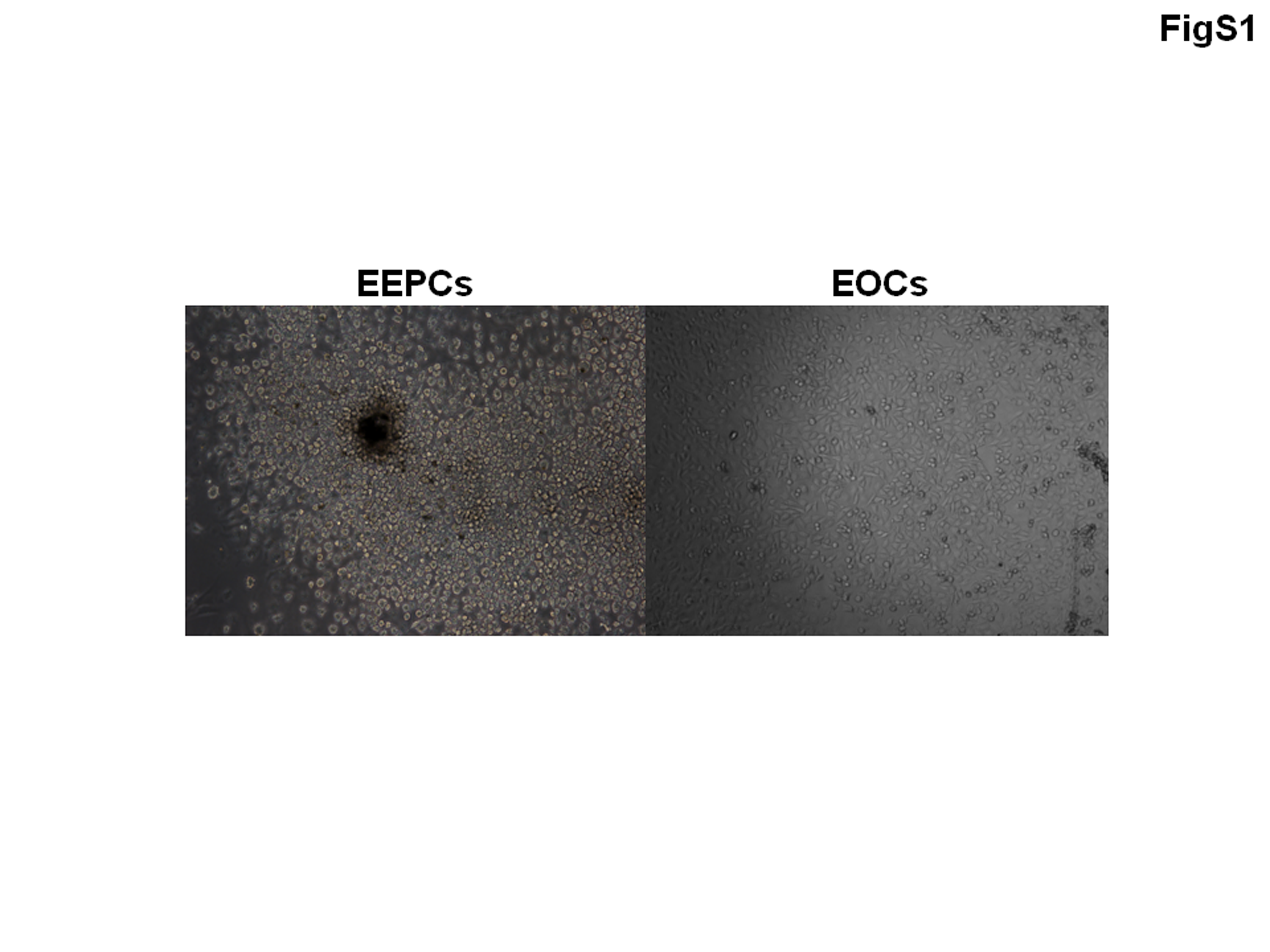

Supplement: Figure S1 — Culture of EEPCs and EOCs. EEPCs and EOCs were cultured as described in Materials and methods, and cells were photographed under a phase-contrast microscope. Magnifications, ×200. (TIF) [file pone.0043643.s001.tif]

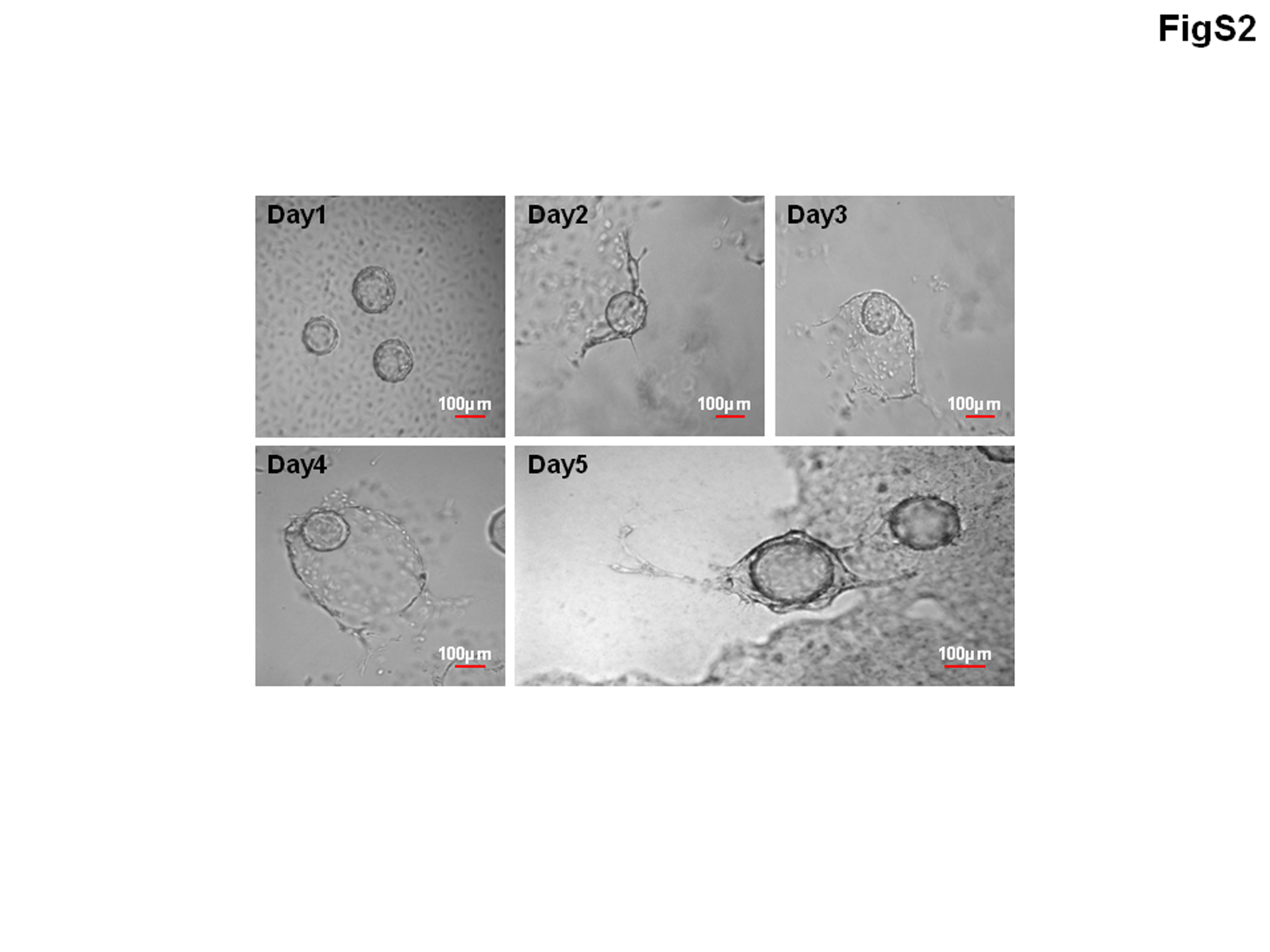

Supplement: Figure S2 — Sprouting and tube formation of EOCs attached to Cytodex 3 microcarrier beads. For methods, see the Materials and methods section of the text. Beads are 70 to 150 µm in diameter. (TIF) [file pone.0043643.s002.tif]

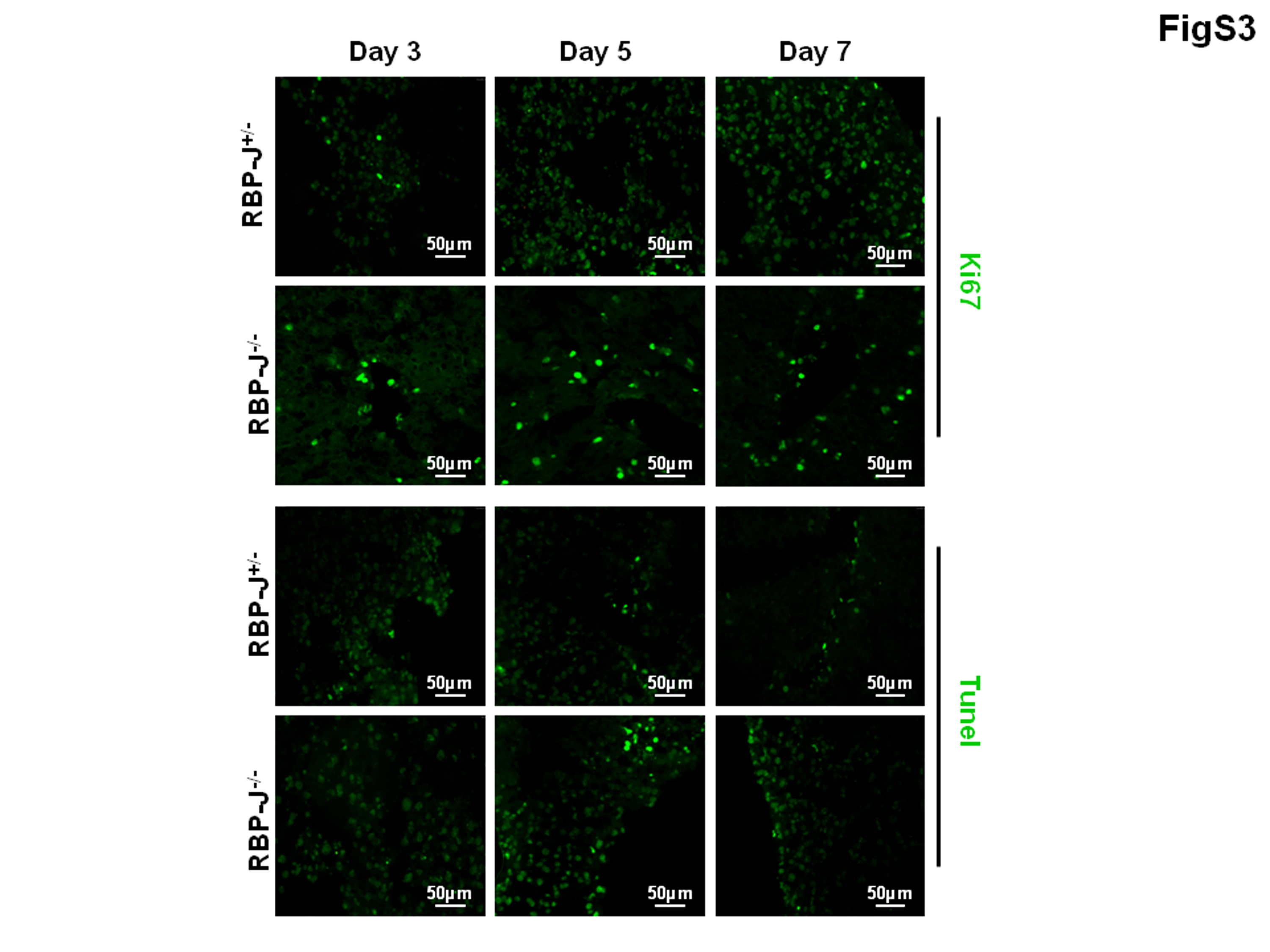

Supplement: Figure S3 — RBP-J deficiency attenuated cell proliferation and increased apoptosis after the transfusion of EEPCs during liver regeneration after PHx. Mice were subjected to PHx and were transfused with EEPCs derived from the RBP-J+/− or the RBP-J−/− mice. Cell proliferation and apoptosis in the livers of the recipient mice was determined on day 3, 5 and 7 after the transfusion by using anti-Ki67 and TUNEL staining, respectively. Ki67+ round nuclei and TUNEL+ cells were counted under microscope. Comparison of the number of cells was shown in Figure 6A. (TIF) [file pone.0043643.s003.tif]

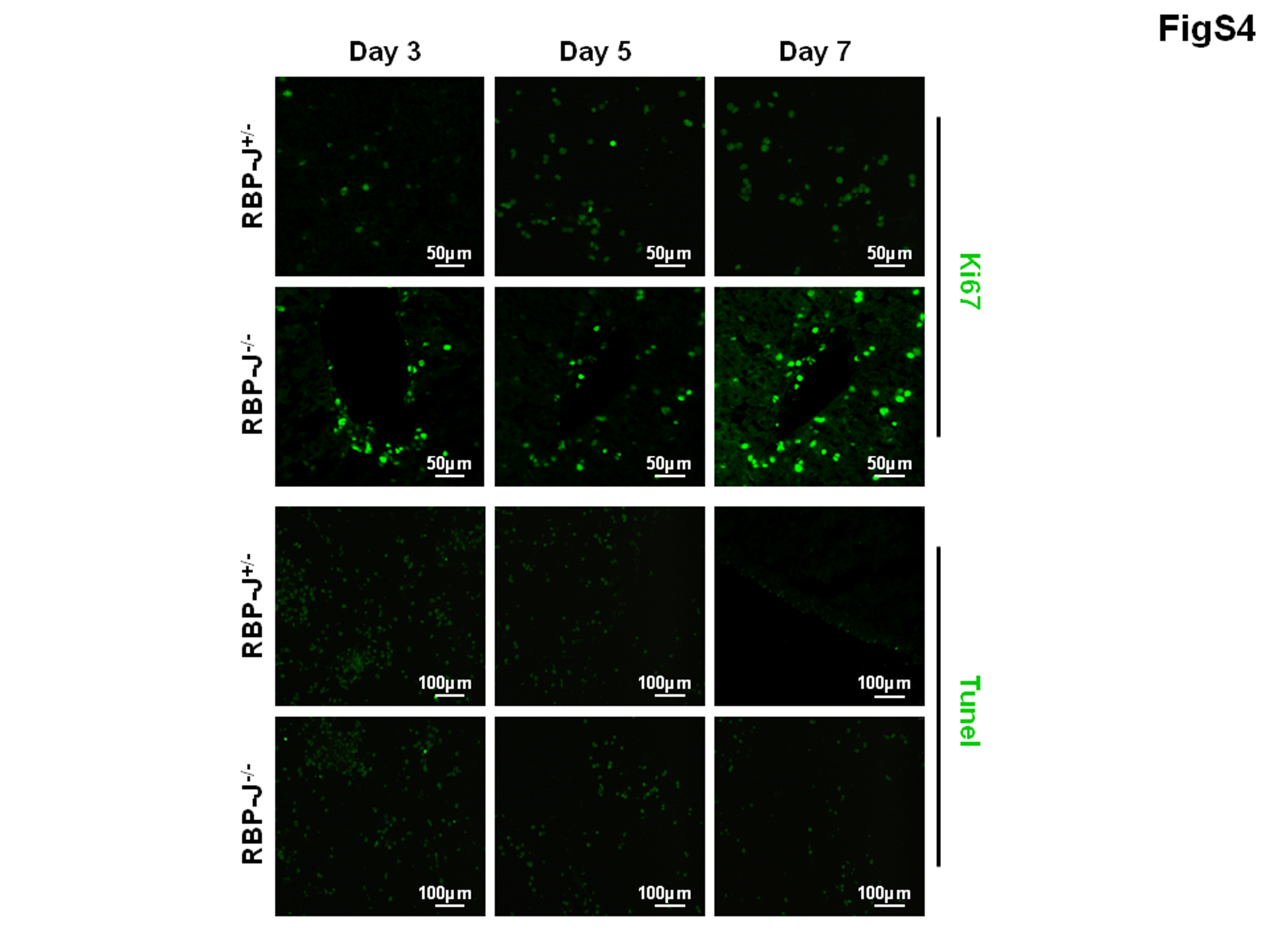

Supplement: Figure S4 — RBP-J deficiency attenuated apoptosis and increased cell proliferation after the transfusion of EOCs during liver regeneration after PHx. Mice were subjected to PHx and were transfused with EOCs derived from the RBP-J+/− or the RBP-J−/− mice. Cell proliferation and apoptosis in the livers of the recipient mice were determined on day 3, 5 and 7 after the transfusion by using anti-Ki67 and TUNEL staining, respectively. Ki67+ round nuclei and TUNEL+ cells were counted under microscope. Comparison of the number of TUNEL+ cells was shown in Figure 6B. (TIF) [file pone.0043643.s004.tif]

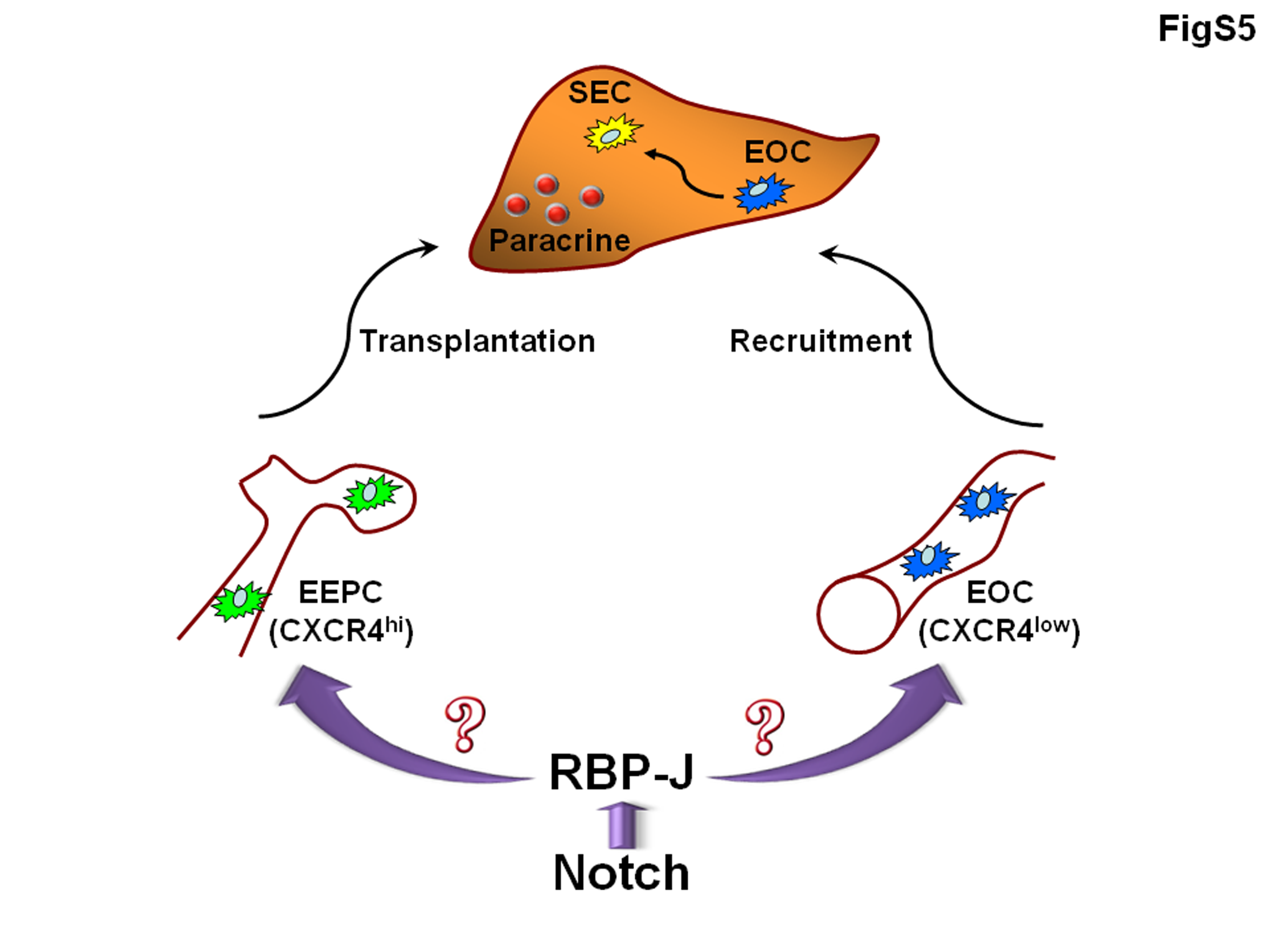

Supplement: Figure S5 — Differential regulations of EEPCs and EOCs by the Notch-CXCR4 signaling. Notch signaling increases homing of EEPCs in BM by the upregulation of CXCR4. In contrast, Notch signaling represses CXCR4 expression by EOCs, therefore reduces their homing to BM. EOCs can be recruited into injured tissues by other signals such as VEGF, and participate in vessel formation likely through vasculogenesis. Therapeutic transfusion of EEPCs can lead to recruitment of EEPCs into injured liver and participates in tissue repair and regeneration through paracrine effects. (TIF) [file pone.0043643.s005.tif]
